# Genetic Analysis of Circadian Responses to Low Frequency Electromagnetic Fields in *Drosophila melanogaster*

**DOI:** 10.1371/journal.pgen.1004804

**Published:** 2014-12-04

**Authors:** Giorgio Fedele, Mathew D. Edwards, Supriya Bhutani, John M. Hares, Manuel Murbach, Edward W. Green, Stephane Dissel, Michael H. Hastings, Ezio Rosato, Charalambos P. Kyriacou

**Affiliations:** 1 Department of Genetics, University of Leicester, Leicester, United Kingdom; 2 Division of Neurobiology, Medical Research Council Laboratory of Molecular Biology, Cambridge, United Kingdom; 3 IT'IS Foundation, Zurich, Switzerland; 4 Institute for Biomedical Engineering, University and ETH Zurich, Zurich, Switzerland; Washington University Medical School, United States of America

## Abstract

The blue-light sensitive photoreceptor cryptochrome (CRY) may act as a magneto-receptor through formation of radical pairs involving a triad of tryptophans. Previous genetic analyses of behavioral responses of *Drosophila* to electromagnetic fields using conditioning, circadian and geotaxis assays have lent some support to the radical pair model (RPM). Here, we describe a new method that generates consistent and reliable circadian responses to electromagnetic fields that differ substantially from those already reported. We used the Schuderer apparatus to isolate *Drosophila* from local environmental variables, and observe extremely low frequency (3 to 50 Hz) field-induced changes in two locomotor phenotypes, circadian period and activity levels. These field-induced phenotypes are CRY- and blue-light dependent, and are correlated with enhanced CRY stability. Mutational analysis of the terminal tryptophan of the triad hypothesised to be indispensable to the electron transfer required by the RPM reveals that this residue is not necessary for field responses. We observe that deletion of the CRY C-terminus dramatically attenuates the EMF-induced period changes, whereas the N-terminus underlies the hyperactivity. Most strikingly, an isolated CRY C-terminus that does not encode the Tryptophan triad nor the FAD binding domain is nevertheless able to mediate a modest EMF-induced period change. Finally, we observe that *hCRY2*, but not *hCRY1*, transformants can detect EMFs, suggesting that *hCRY2* is blue light-responsive. In contrast, when we examined circadian molecular cycles in wild-type mouse suprachiasmatic nuclei slices under blue light, there was no field effect. Our results are therefore not consistent with the classical Trp triad-mediated RPM and suggest that CRYs act as blue-light/EMF sensors depending on trans-acting factors that are present in particular cellular environments.

## Author Summary

Low frequency electromagnetic fields (EMFs) are associated with electrical power lines and have been implicated in the development of childhood leukemias. However, the Earth also has a natural EMF that animals can detect and which they use in order to navigate and orient themselves, particularly during migrations. One way they might do this is by using specialised photoreceptors called cryptochromes, which when activated by light, generate changes within the molecule that are susceptible to EMFs. Cryptochromes are important components of animal circadian clocks, the 24 hour timers that determine daily behavioral and physiological cycles. We have studied the circadian behavior of the fruitfly and have observed some novel and robust effects of EMFs on the fly's sleep-wake cycle that are mediated by cryptochrome. By using cryptochrome mutants we find that our results do not support the classic model for how this molecule might respond to EMFs. We also show that mammalian cryptochromes can respond to EMF when placed into transgenic Drosophila, whereas in mammalian clock neurons, they cannot. Consequently, the EMF responsiveness of cryptochrome is determined by its intracellular environment, suggesting that other, unknown molecules that interact with cryptochrome are also very important.

## Introduction

A wide range of animals are able to detect and exploit the Earth's magnetic field, particularly for the purposes of orientation and navigation [1]–[3]. The biological basis for the detection of electromagnetic fields (EMFs) is not understood but two main theories have been presented. The first involves crystals of magnetite (iron oxide, Fe_3_O_4_) that can be found in the upper beaks of birds [4] or in the nasal regions of salmonid fish [5]. The second suggests that photoreceptors may play a significant role through the radical pair mechanism (RPM) whereby biochemical reactions generate radical pairs that become sensitive to EMFs [6].

One class of photoreceptors that meets the requirements for the RPM is cryptochrome (CRY), a blue-light photoreceptor that in *Arabidopsis* is proposed to mediate the effects of EMFs through electron transfer between a triad of Tryptophan residues and the flavin cofactor FAD [7], [8]. In *Drosophila melanogaster*, CRY is the deep-brain photoreceptor that mediates circadian responses to light [9]–[11], making it a suitable model for studying any link between circadian clock and magnetoreception. In non-drosophilid insects, there can be two CRY homologues, one which plays the circadian photoreceptor role, type 1 CRY, and another, type 2, that acts as the main negative autoregulator for the circadian clock and does not apparently respond to light [12], [13]. In mammals, there are no Type 1 CRYs but two paralogues of Type 2 CRY, which both act as negative autoregulators of the circadian clock [14], [15], but can retain light responsiveness under some conditions [16].

*D. melanogaster* responds to low intensity EMFs under wavelengths of light to which CRYs are sensitive, but the adaptive implications of these magnetic effects on fly orientation are unclear [17]–[19]. Recently, the genetic and molecular basis of fly magneto-sensitivity has been explored using four different experimental paradigms that have converged on the finding that CRY plays a key role in the EMF response [20], [21], [29]. In the first paradigm, naïve responses of populations of flies to a static EMF are enhanced by associating the field with sucrose and this conditioned response is eliminated in *cry* mutants [20]. Mutagenesis of tryptophan within the triad (residues Trp-342, Trp-397 and Trp-420 in *Drosophila* CRY) in the FAD chromophore domain, however, did not disrupt the ability of type 1 *cry* transgenes from the Monarch butterfly or *Drosophila* to rescue the EMF response in *cry-null* mutants [22] Thus it may be that a mechanism other than radical pairs involving the Trp triad is used by Type 1 CRY molecules to sense EMFs. Indeed superoxide radicals and ascorbic acid have been proposed as suitable candidates for forming a radical pair with the FAD [23], [24]. Furthermore, Type 2 human *hCRY2* was also able to rescue the fly's EMF response in blue light, suggesting that in a *Drosophila* cellular environment, hCRY2 may be photosensitive [25].

In the second paradigm, responses to EMF are explicitly clock-dependent and rely on the observation that in constant dim blue light (LL), circadian periods are usually significantly lengthened beyond 24 h due to constitutive activation of CRY [26]. On applying a static EMF for a number of days, about 50% of wild-type flies either lengthened or shortened their circadian period [21]. This alteration in period on EMF exposure is not observed in *cry* mutants, but as the initial period lengthening due to dim blue light is CRY-dependent, there is no period change for the subsequent EMF exposure to modify. Nevertheless, a relevant observation from this study is that overexpression of CRY in clock neurons leads to a significant decrease in rhythmicity and a variable enhancement of the period changes during EMF exposure in the few animals that were reported to remain rhythmic under these conditions [21]. In both the conditioning and circadian paradigms, the sensing of EMF by flies is wavelength dependent and focused on the action spectra and absorption characteristics of CRY, which is in the blue and UV range [20], [21].

The third paradigm, involves negative geotaxis of adult flies, and is the fly's tendency to walk upwards against gravity. This phenotype is CRY mediated [27], [28] and is susceptible to disruption by static EMFs under blue light [29]. In addition, key CRY-expressing structures such as the eyes, the antennae and a subset of circadian clock neurons, contribute to the EMF geotactic phenotype [29]. The fourth paradigm involves a CRY-mediated increase in the recovery time of *Drosophila* larvae from electric shock when they are exposed to a static EMF under blue light [30]. In our study we sought to re-examine the effects of EMF on circadian behavior using the Schuderer apparatus, in which responses to EMF can be studied without interference from the Earth's natural magnetic field or from other local magnetic/radiofrequency fields [31]. Under these more controlled and stringent conditions, there is a highly robust and consistent CRY-dependent period response to extremely low frequency and static EMFs as well as an additional novel locomotor phenotype. Further use of *cry* variants reveals some surprising results, which are difficult to explain with the current RPM. Finally we reveal that the cellular environment of mammalian CRY2 determines whether it is light-sensitive and can respond to EMFs, suggesting that trans-acting factors are critical for CRYs mediation of field effects.

## Results

**Figure 1:**
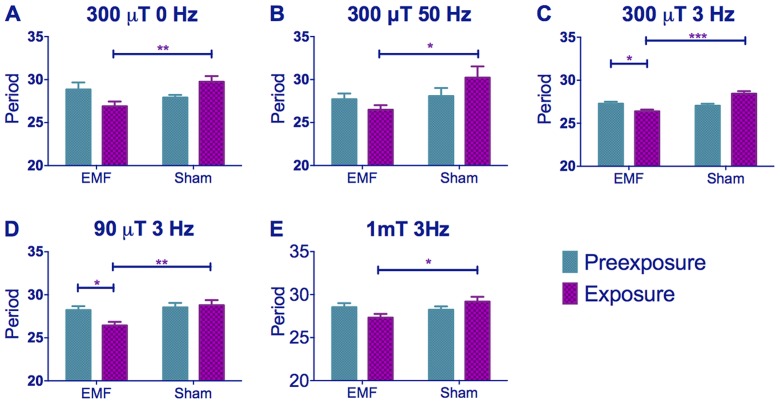
EMF exposure shortens free-running circadian periods in dim blue light. Mean circadian periods (h) +/− sem are shown for the EMF and sham-exposed groups. Note how periods are considerably longer than 24 h. (A–C) period changes in CS flies under static, 50 and 3 Hz field respectively at 300 µT (C–E) period changes in CS flies under 300, 90 and 1000 µT (1 mT) field respectively at 3 Hz. EMF-exposed flies show significant period shortening. For period and N see Table S1. (*post-hoc* *p<0.05, **p<0.01, ***p<0.001).

We primarily used 300 µT for our experiments, as this was the intensity used in Yoshii *et al.*, (2009), but we also studied two additional intensities, 90 µT (closer to the Earth's ambient magnetic field) and 1 mT (1000 µT). The minimum frequency possible in the Schuderer apparatus was initially 3 Hz [31] but we also tested 50 Hz (the common frequency in Europe). A subsequent upgrade of the equipment allowed us to also test a static field. Thus the frequencies we used fell within the range of background frequency called the Schumann Resonance [32]. The experimental design was as follows: two groups of flies of the same genotype were studied for seven days under constant dim blue light (LL, hereafter termed pre-exposure) followed by eight days under the same illumination but exposed either to an EMF (EMF exposure) or a sham EMF (sham exposure). The circadian locomotor period was then calculated separately for the pre-exposure and exposure days for each fly and compared (see Methods section for more details). We examined the EMF responses of flies using a standard field intensity of 300 µT with stationary, 3 Hz or 50 Hz frequencies (Figure 1A–C), or using a standard 3 Hz frequency with field intensities of 90, 300 or 1000 µT (1 mT, Figure 1C–E). Irrespective of frequency or intensity of the field, sham-exposed Canton-S (CS) exhibited a lengthening in period between the initial LL pre-exposure and the sham exposure due to the constitutive activation of CRY [26], whereas the EMF-exposed flies showed a significantly shorter period compared to the corresponding sham-exposed flies and to their own pre-exposure (Figure 1, 2A). A three way ANOVA revealed significant effects for EMF frequency (F_(2,294)_ = 37.28, p∼0), exposure to EMF/sham (F_(1,294)_ = 14.81, p<0.001), and for the two-way interaction between pre-exposure and EMF/sham (F_(1,294)_ = 21.73, p<0.01). Importantly, there was no significant three-way interaction (F_(2,294)_ = 1.01, p = 0.36), revealing that a similar pattern is revealed at all three frequencies at 300 µT (Figure 1A–C). Three way ANOVA also revealed significant effects for intensity (F_(2, 272)_ = 23.59, p<0.001) exposure to EMF/sham (F_(1,272)_ = 16.69, p<0.001) and for the pre-exposure x EMF/sham interaction (F_(1, 272)_ = 19.38, p<0.001). There was no significant 3-way interaction (F_(2, 272)_ = 0.04, p = 0.96) showing that the flies were responding in a similar manner to these exposures at 3 Hz (Figure 1C–E, Table S1).

**Figure 2:**
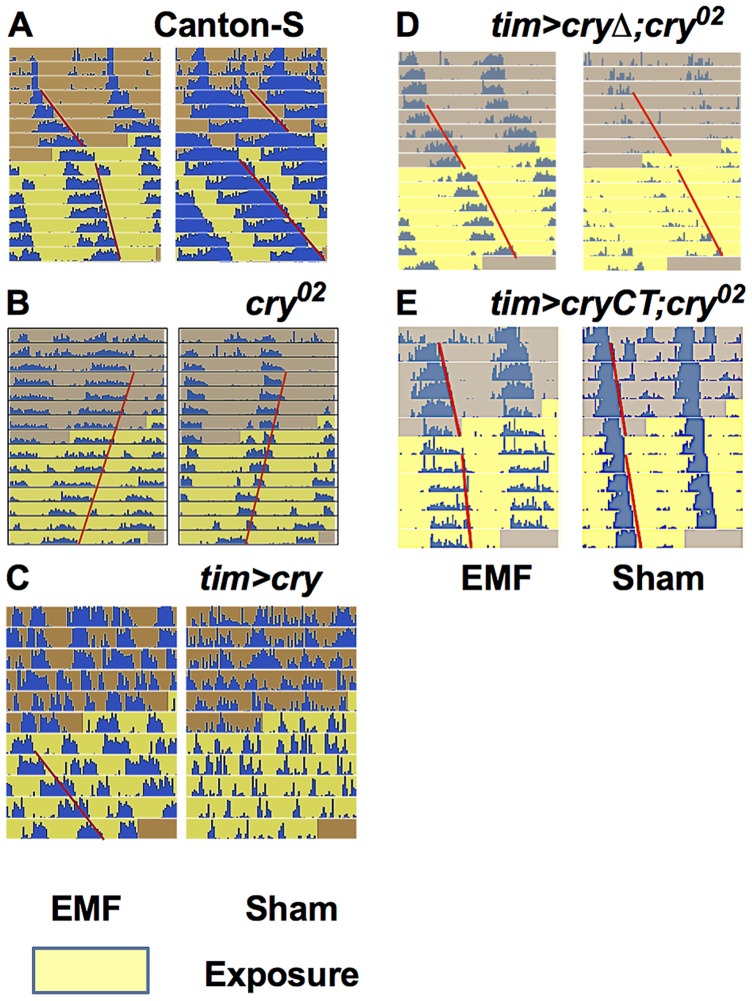
EMF exposure shortens circadian period. Representative free-running locomotor rhythms in dim blue, constant LL before and during the exposure to EMF (300 µT, 3 Hz). A. Exposed Canton-S flies showed a significant period shortening compared to sham. B. *cry^02^* flies did not show any EMF effect and maintain their free-run during the exposure period. C. Most exposed *tim>cry* flies showed arrhythmia before, but a well-defined period during the EMF exposure. D. *tim>cryΔ;cry^02^* are not EMF sensitive. E. *tim>cryCT;cry^02^* show an EMF effect with a slight period shortening compared to sham exposed flies. Each horizontal line show activity events (blue) double plotted for two successive 24 hour periods, day 1 and 2 on the top line, day 2 and 3 on the second line and so on. The red line outlines the activity offset.

To study whether any of these effects associated with EMF exposure could be due to artefacts, particularly those caused by any vibration produced by the electric current flowing through the coils or the turning of the fans in each chamber, we performed a number of additional control experiments. However, manipulating the putative sources of vibration did not reveal any effects that could have contributed to our behavioral results (Figure S1).

**Figure 3:**
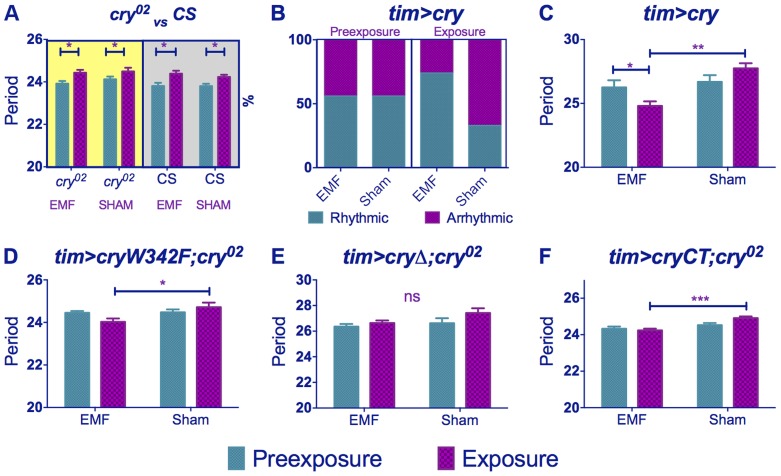
*cry* variants alter normal circadian responses to EMFs. Circadian periods (h) in dim blue LL are shown for EMF and sham-exposed groups. Mean periods ± sem. (A) *cry^02^* flies exposed to EMF show only ageing effects on period (yellow shaded box). Wild-type flies kept in DD (grey shaded box) show similar ageing effects (B) *tim>cry* % rhythmic/arrhythmic flies during pre-exposure and exposure to EMF or sham. Exposure to EMF dramatically increases the proportion of rhythmic flies (χ^2^_(3)_ = 12.78, p<0.01). (C) *tim>cry* period for EMF exposed and sham flies before and during exposure (D) *tim>cryW342F;cry^02^* (E) *tim>cryΔ;cry^02^*. (F) *tim>GFPcryCT;cry^02^*. (See Table S1, *post-hoc* *p<0.05, **p<0.01, ***p<0.001).

We therefore pursued our analyses using a 3 Hz/300 µT EMF to study any effect of the *cry^02^* null mutation [33]. The response to the EMF was abolished in *cry^02^* flies (Figure 2B, 3A, Table S1), consistent with a possible role for CRY in determining this phenotype (pre-exposure x EMF/sham exposure interaction F_(1,52)_ = 2.93, p = 0.09). However, as mentioned earlier, CRY is required in order to generate the initial blue light-dependent lengthening of period and so these results are not informative in determining whether CRY is the magnetoreceptor. *cry^02^* flies did show a slight lengthening of period between the pre- and exposure conditions of about 0.5 h (F_(1,52)_ = 108.4, p<0.001, Table S1) suggesting an ageing effect over the ∼15 day observation [28]. Indeed we observed a similar period lengthening in CS flies exposed to DD for the same number of days during which CRY would not be light-activated (F_(1,54)_ = 14.40, p<0.001, Figure 3A, Table S1). ANOVA revealed no significant three-way interaction when we compared CS in DD to *cry^02^* in LL (genotype x pre-exposure x EMF/sham exposure, F _(2, 106)_ = 0.07, p = 0.79), supporting the view that the slight lengthening of period was due to ageing. This experiment also clearly shows how the period-shortening of CS flies under EMF is light-dependent (Compare Figure 3A in DD with Figure 1C). Consequently the more dramatic lengthening in period of 1–2 h (Figure 1A–E) observed in CS flies in sham conditions under dim blue LL will also include a small ageing component in addition to that generated by constitutive CRY expression (Table S1). The shortening of period in wild-type flies exposed to EMF is therefore observed in spite of a natural tendency of the flies to increase their period over the duration of the experiment due to ageing (Figure 1, Table S1).

We then overexpressed *cry* in clock cells using *timgal4* and observed that ∼55% of the *timgal4>cry* flies in the wild-type background became arrhythmic during the initial LL pre-exposure interval, consistent with a hyper-activation of CRY (Figure 2C, 3B, Table S1). EMF-exposure, however, abrogated arrhythmicity to ∼25%, suggesting a disruption of CRY signalling under these conditions, whereas sham-exposed flies showed 67% arrhythmicity (χ^2^_(3)_ = 13.96, p<0.05, Figure 3B, 2C, Table S1). Furthermore, the flies that stayed rhythmic throughout the *timgal4>cry* experiment again revealed a significant shortening in period under EMF compared to the sham controls (pre-exposure x EMF/sham exposure interaction (F_(1,79)_ = 6.23, p = 0.015, Figure 3C, Table S1).

We next examined the responses of the *UAScryW342F* mutant under *timgal4* control in a *cry^02^* background (Figure S2) [22]. This mutant carries a Trp to Phe substitution in the final Trp forming the Trp triad that is responsible for donating the required electron to the cascade during light activation [34]. Nevertheless, this mutant is light responsive and significantly lengthens its period in dim blue light (Figure S3A, Table S1). We observed a significant period shortening in EMF exposed compared to sham flies (pre-exposure x EMF/sham exposure interaction F _(1,54)_ = 4.15, p<0.05, Figure 3D, Table S1). Consequently mutation of Trp-342 in the triad believed to be necessary for the RPM does not significantly disrupt the circadian response to EMF.

We also used the *UAScryΔ* mutation (Figure S2), again under control of *timgal4*, in which residues 521–540 of the C-terminal have been deleted [26]. *timgal4>cryΔ* flies have a long free-running period in DD as if CRY is constitutively active, but CRYΔ can be further activated by blue light [26], [35]. We confirmed this observation by showing that flies carrying *timgal4>cryΔ* in a *cry*-null background showed a lengthening of period of 1.2 h under dim blue light compared to DD (F_(1,34)_ = 6.53, p<0.01, Figure S3B). Surprisingly, however, they did not show any significant period changes under EMF exposure (pre-exposure x EMF/sham Exposure F_(1,174)_ = 0.74, p = 0.39, Figure 2D, 3E, Table S1) implicating the C-terminal of CRY (CT) in the response to EMF. We therefore tested flies expressing a GFP-CRY-CT (Figure S2) fusion in a *cry^02^* genetic background (*UASGFPcryCT;timGAL4;cry^02^*). This construct carries only the CRY C-terminal residues 491–542 fused to GFP (see Methods). Remarkably, these flies were still able to respond to light (Figure S3C) and also show a modest response to the EMF (F_(1,118)_ = 4.9, p<0.02; Figure 2E, 3F, Table S1) confirming the importance of the CRY-CT in the EMF response. We also performed the same experiment in DD but we did not observe any significant EMF effect (pre-exposure x EMF/sham exposure F_(1,82)_ = 0.1, p = 0.81) although we did find the ageing effect on period (pre-exposure vs exposure F_(1,82)_ = 4.2, p<0.05). Consequently, for *UASGFPcryCT;timGAL4;cry^02^* flies, the slight reduction in period between the pre- and EMF exposure occurs in spite of the ageing effect which would tend to increase period between the two conditions. We should also note here that pre-exposed *UASGFPcryCT;timGAL4;cry^02^* flies have periods very close to 24 h and only 0.4 h longer than their DD controls (Table S1), so there is little room to reduce this period further given that CRY is not a canonical clock molecule. Consequently, it would be difficult to see how any CRY manipulation could yield periods shorter than the DD free-running period via changes in CRYs light-mediated TIM interactions and consequent input to the clock.

### A novel locomotor phenotype is sensitive to EMF

**Figure 4:**
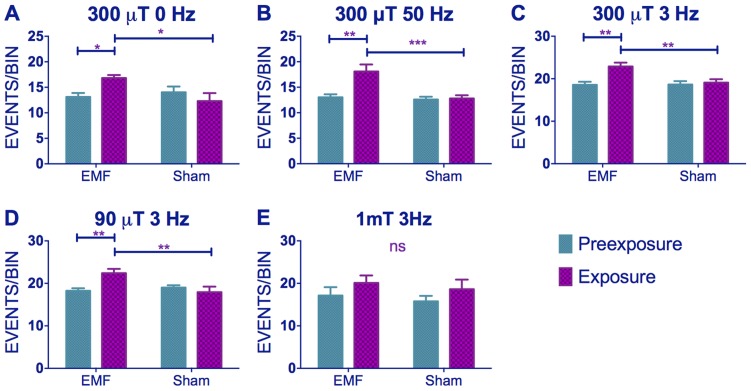
EMFs increase activity levels in wild-type flies. (A–C) Hyperactivity in EMF-exposed CS under static, 50 and 3 Hz field respectively at 300 µT. (C–E) Hyperactivity in CS flies under 300, 90 and 1000 µT field respectively at 3 Hz. N's are the same as in Figure 1. Mean activity events per 30 min time bin (± sem). For average activity and N refer to Table S2 (*post-hoc* *p<0.05, **p<0.01, ***p<0.001).

When we scrutinised further our locomotor activity records we observed that exposure to low frequency EMF not only shortened circadian period but it also caused significant hyperactivity in wild-type flies. Comparison of static to 3 and 50 Hz at 300 µT fields revealed significant Frequency (F_(2,294)_ = 42.35, p∼0), sham/EMF F_(1,294)_ = 6.75, p<0.01), pre-exposure/exposure (F_(1,294)_ = 7.98, p<0.01) and pre-exposure x EMF/sham exposure interaction (F_(1,294)_ = 7.93, p<0.001), but no significant three-way interaction (F_(2,294)_ = 0.17, p = 0.83) illustrating that all frequencies gave a similar pattern of EMF mediated hyperactivity (Figure 4A–C, Table S2). When we compared 90, 300 and 1000 µT at 3 Hz we did not observe a significant Intensity effect (F_(2,272)_ = 2.14, p = 0.1), but sham/EMF (F_(1,272)_ = 4.66 p<0.05), pre-exposure/exposure (F_(1,272)_ = 8.133, p<0.05) and pre-exposure x EMF/sham exposure interactions (F_(1,2272_ = 3.71, p = 0.05) were all significant (Figure 4C–E, Table S2). *Post-hoc* tests revealed a significant hyperactivity in EMF exposed flies compared to sham at 90 and 300 µT, but not at 1 mT, but this difference was not sufficient to generate a significant three-way interaction (F_(2,272)_ = 0.71, p = 0.5).

**Figure 5:**
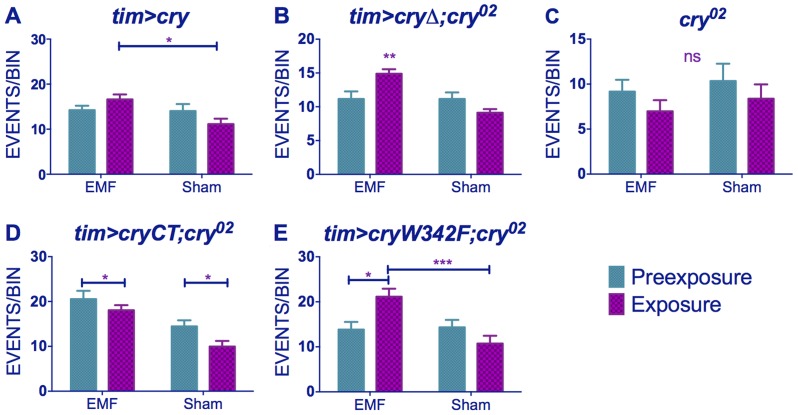
EMF-induced hyperactivity in *cry* variants. (A) *tim>cry* (B) *tim>cryΔ;cry^02^* (C) *cry^02^* (D) *tim>cryCT;cry^02^* (E) *tim>cryW342 F;cry^02^* N's are the same as in Figure 3. Mean ± sem. (see Table S2, *post-hoc* *p<0.05, **p<0.01, ***p<0.001).

Similar results were obtained for *timgal4>cry* overexpressing flies (pre-exposure x EMF/sham exposure interaction (F_(1,79)_ = 4.021, p<0.05, Figure 5A, Table S2) revealing that EMF-exposed flies showed enhanced hyperactivity compared to sham and pre-exposed flies. More surprisingly, *timgal4>cryΔ* flies also expressed this hyperactivity under EMF exposure (pre-exposure x EMF/sham Exposure interaction F _(1,174)_ = 11.28, p<0.01, Figure 5B, Table S2) whereas no locomotor differences were detected in *cry^02^* (pre-exposure x EMF/sham exposure interaction, F_(1, 52)_ = 0.04, p = 0.95, Figure 5C,Table S2) nor in *UASGFPcryCT;timGAL4;cry^02^* (pre-exposure x EMF/sham interaction, F_(1, 118)_ = 0.51, p = 0.46, Figure 5D, Table S2). Furthermore flies expressing the *cryW342F* mutation also exhibited the hyperactivity associated with EMF exposure (F_(1,54)_ = 11.9 p<0.01, Figure 5E, Table S2). We therefore conclude that while robust EMF-induced shortening of circadian period requires the CRY C-terminus, the hyperactivity appears to be determined via the N-terminal photolyase-like domain and is not susceptible to disruption by the Trp-342 mutation, indicating that alternative routes are available for the RPM.

### hCRY and magnetoreception

**Figure 6:**
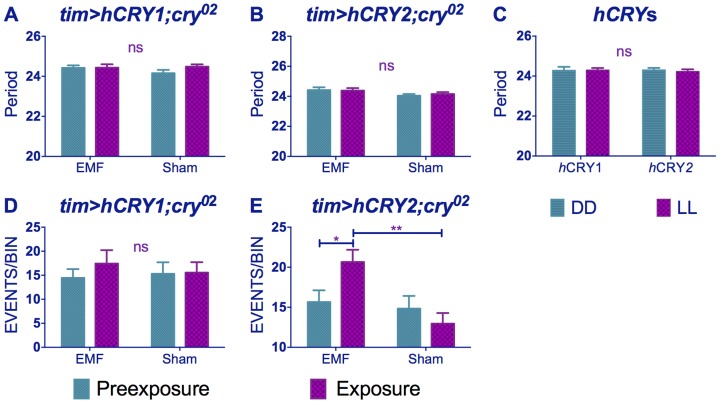
*hCRY2* but not *hCRY1* reveals a sensitivity to EMFs. (A) *tim>hCRY1; cry^02^* or (B) *tim>hCRY2; cry^02^* transformants do not show period shortening under EMF (pre-exposure*EMF/sham interaction *hCRY1* F_(1,48)_ = 1.41, p = 0.3 *hCRY2* F_(1,54)_ = 0.2, p = 0.63 (see Table S1). (C) *hCRY1/2* flies do not show period increase in dim blue LL compared to DD (F_(1, 82)_ = 0.125, p = 0.72) (D) *hCRY1* are not hyperactive under EMF (F_(1,48)_ = 0.33, p = 0.56). (E) *hCRY2* are hyperactive under EMF exposure. Mean ± sem (see Table S2, *post hoc* * = p<0.05, ** = p<0.01).

Flies expressing vertebrate non-photoreceptor *hCRY2* are reported to exhibit light-dependent magnetoreception in a conditioning assay [25]. By separately expressing *tim-GAL4>hCRY1* or *hCRY2* on a *cry^02^* background, we observed no significant differences in period between exposed and sham flies (Figure 6A, B, Table S1). Indeed, the *hCRY1/*2 flies behaved as if they did not respond to dim blue LL because their circadian period does not lengthen in LL compared to DD (Figure 6C), although hCRY proteins have been shown to be light degraded in flies [16] (Fig. S3) and *hCRY2* has been implicated in mediating EMF response in a light dependent manner [25]. Nevertheless and somewhat surprisingly, flies expressing *hCRY2* but not *hCRY1* showed the EMF-induced hyperactivity phenotype (*hCRY2* pre-exposure x sham interaction F_(1,54)_ = 5.69 p<0.05, Figure 6D, E, Table S2).

### *Drosophila* CRY is stabilised by EMF

**Figure 7:**
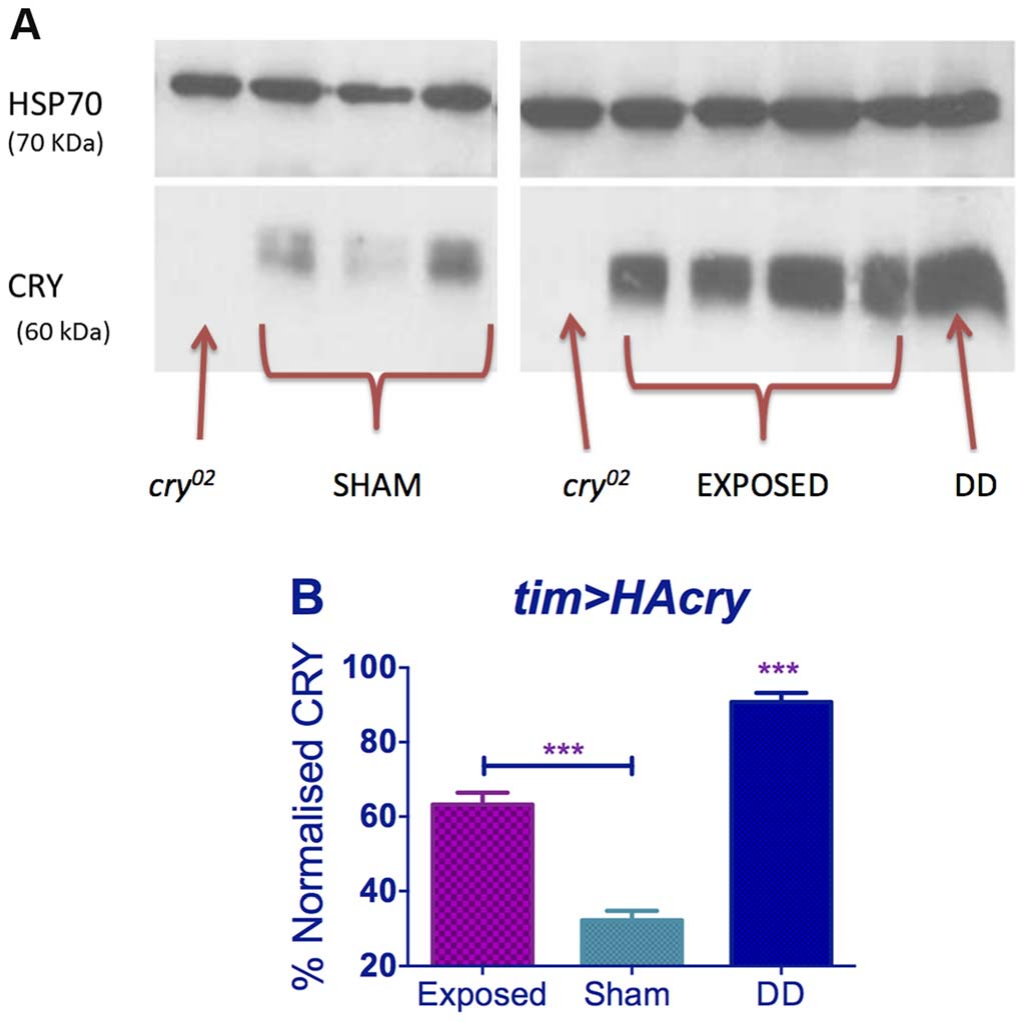
EMF exposure increases CRY stability. Top panel. Western blots for CRY using anti-dCRY in wild-type flies expose to EMF or sham in dim blue LL with *cry^02^* and DD control. HSP is used as loading control. Bottom panel. Quantification based on 3 biological replicates each with 3 technical replicates (repeated measures ANOVA F_(2,6)_ = 113.1, p<0.001, *post hoc* *** p<0.001). Mean ± sem.

Western analysis revealed, that levels of CRY in DD were significantly elevated compared to sham in dim blue light as expected [11], but we also observed that under EMF exposure, CRY was significantly more abundant compared to sham (p<0.001, Figure 7). EMF therefore appears to reduce CRY degradation, which in turn would suggest that CRY signalling is compromised.

### Molecular circadian rhythms in mouse SCN slices do not respond to EMFs

Given that the EMF hyperactivity response could be rescued in fly transformants carrying *hCRY2*, we asked whether mammalian type 2 CRYs could also be EMF responsive in a circadian context. We therefore used the Schuderer apparatus to expose SCN slices to EMFs ranging from 50 to 500 µT at 50 Hz and examined the rhythmic bioluminescence of the PER2::LUC reporter (Figure S4A, B). We have shown previously that these rhythms are dependent on CRY1 and CRY2 [36] SCN slices were housed in exposure chambers for 5 days with field exposure strengths of 50, 150, 300 and 500 µT, followed by 5 days in sham conditions of 0 µT or *vice versa* in a paired crossover design. All slices generated very clear and sustained circadian cycles of bioluminescence (Figure S4B). No significant differences were observed, however, in period, period error or relative amplitude error (see Methods) between exposed and sham conditions under any of the EMF intensities (Figure S4 C–H). We also compared the effects of blue versus red light with a 300 µT, 50 Hz field, but again, no significant differences in the three rhythm measures were observed between sham and EMF exposed slices (Figure S5). Thus, if mammalian CRY1 and/or CRY2 have the intrinsic capacity to mediate light-dependent sensing of EMF, the specific CRY-dependent response and/or the intracellular context of the protein may be critical in determining its function.

## Discussion

We have identified two light-dependent and robust behavioral responses to EMF in the fly; shortening of circadian period and locomotor hyperactivity. Our findings are consistent with an underlying CRY-dependent magneto-response and importantly confirm and extend the most relevant observation of Yoshii et al (2009), which was that overexpression of CRY in clock neurons enhances the circadian response to EMF. This was observed in two ways in our study, by an increase in the proportion of rhythmicity under EMF in flies overexpressing CRY (55 *v.* 76%) as well as in an enhanced shortening of circadian period between sham- and EMF-exposed conditions of wild-type versus CRY overexpressing flies (2.07 h±0.34 versus 2.95 h±0.75, respectively Figure 1C, 3C, Table S1). However, these results contrast sharply with those of Yoshii *et al* [21], who observed a significant *decrease* in the proportion of rhythmic CRY-overexpressing flies under EMF and a predominant *lengthening* of period. While both sets of results indirectly support the role of CRY in magnetosensitivity it is unlikely that these differences are solely due to the more controlled EMF environment generated by the Schuderer apparatus.

**Figure 8:**
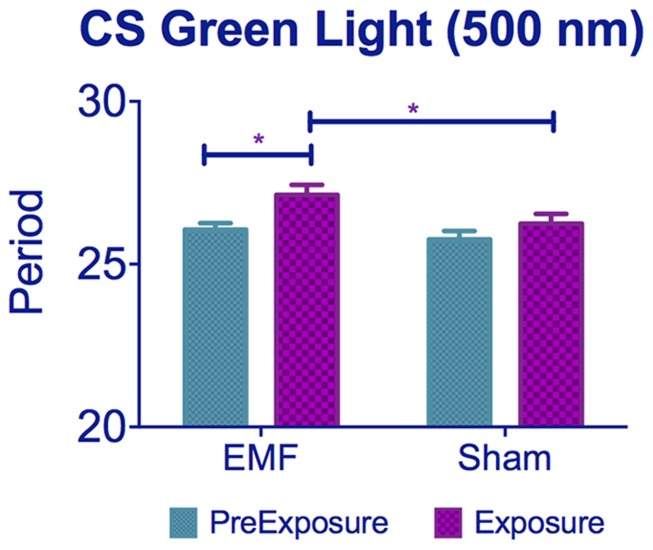
Exposure to 500 nm green light lengthens circadian period under EMF. CS flies kept under 500 nm show period lengthening when exposed to EMF compared to sham flies. See Table S1, *post-hoc* *p<0.05, ***p<0.001). Mean ± sem.

This contradiction may conceivably be resolved by considering the action spectrum of CRY [16], [37] and the ‘antagonistic effect’ of the magnetic field in response to light [38], [39]. Under this proposal, the alignment of the magnetic field would produce inverse or complementary responses under different wavelengths that are dependent on the initial ratio of singlet-triplet states of the radical. This antagonistic effect of wavelength was observed in experiments on magnetic compass orientation in *Drosophila*, which under green light (500 nm) showed a 90° shift in their alignment compared to flies tested under violet light (365 nm) [18]. This wavelength-dependent effect was also proposed to explain why in the EMF conditioning experiments of Gegear *et al.* (2008), flies failed to exhibit a response to EMF under full spectrum light when wavelengths below 420 nm were filtered out [38]. As pointed out by Phillips and co-workers, this failure could be due to a change in the nature of the response rather than an inability of the flies to sense the field. Indeed, the response of naïve flies to EMF under full spectrum and full spectrum >420 nm has opposite directions [20]. However, the wavelengths used in our study (430–470 nm) compared to the previous work (445–495 nm [21] and Helfrich-Forster, pers comm)) would initially not appear to be sufficiently different to engage any such antagonistic effect, so the opposite features of the results of the two studies remains puzzling. In an attempt to solve this conundrum, we exposed flies to 500 nm (+/−20 nm) in the Schuderer apparatus, and were surprised to observe that EMF exposed flies revealed a *period lengthening* rather than the period-shortening we had observed at 450 nm (EMF/Sham Exposure F _(1,141)_ = 5.12, p<0.05 and pre-exposure/exposure F_(1,141)_ = 8.77, p<0.01, Figure 8). Taken together these results at the different wavelengths favor the RPM and the antagonistic model mentioned above, whereby small changes in wavelengths may result in a different Triplet-Singlet ratio and therefore the S-T interconversions would strongly affect the CRY product yield [38]. This striking result nicely explains why the results of Yoshii *et al.* (2009) are in the opposite direction to ours.

Dim LL lengthens circadian period because activation of CRY alters PER and TIM dynamics, so that nuclear accumulation of these proteins is delayed in s-LNv pacemaker neurons, generating a longer period [26]. The shortening of circadian period observed under EMF thus suggests a partial inactivation of CRY. This interpretation is strongly supported by the results of the western blots, which showed a more stable/abundant CRY under EMF. Upon light absorption, CRY undergoes conformational changes leading to its activation and ultimately to its degradation, which is mediated by E3-ubiquitin ligases [9], [11], [35], [40], [41]. Displacement of the CRY C-terminal (CT) induced by light may increase the binding affinity of CRY to its partners, generating more extended positively and negatively charged regions [42]. Thus significantly more abundant CRY under EMF is likely to be due to CRY maintaining a more inactive conformation that attenuates its light-mediated degradation and prevents period-lengthening [43].

The Trp triad has for some years been considered to be indispensable for the photo-induction of CRY by electron transfer to the FAD, and in the Drosophila CRY structure, these are Trp342, Trp397 and Trp420 [42], [44], [45]. A further residue, Trp536 was initially suggested to lie near the FAD binding pocket, potentially representing an electron donor [44] but more recent dCRY structural analyses have residue Phe534 at this location [42], [45]. Nevertheless double mutant W397F/W536F proteins remain photo-inducible as measured by light induced proteolysis in a cell assay [46]. In addition, the W397F CRY mutant protein was effective in light induced TIM proteolysis even at fluences that do not photoreduce flavin [46]. Furthermore, the redox state of flavin played no significant role in light induced CRY conformational changes nor in downstream interactions with JET (but see [43]). These startling results reveal that photoreduction of flavin may not be the primary mechanism that provides CRY light signalling, even though FAD binding is essential [46]. These results have clear implications for the RPM and provide a rationale for why the W342F mutant retains EMF sensitivity in both our circadian and the conditioning assay. However we should add that there is considerable debate at present on the relevance of the redox status of FAD for CRY light signalling [37], [42], [43], [46], [47]. We also cannot exclude the possibility that another residue such as tyrosine may complete the electron transfer [48], or that a photolyase-like photocycle could be involved [35], [47].

The use of the CRYΔ construct allowed us to decouple the two phenotypic effects of EMF. The period-shortening is significantly attenuated by deletion of the CRY C-terminal, whereas the hyperactivity can be mediated by the N-terminal sequences. According to recent structural analyses of dCRY [42], the deletion of Cys523 in CRYΔ could conceivably alter the photoreduction state of the FAD via Met421 which lies close to Trp397 thereby disrupting electron transfer and, presumably, the EMF-induced period-shortening phenotype. Yet CRYΔ leaves the hyperactivity phenotype intact, suggesting that period-shortening might be more sensitive to disruption of the RPM than hyperactivity. However, this is unlikely because the GFP-CRY-CT construct was competent for inducing modest but significant EMF-induced period shortening compared to its corresponding sham control, if not to the pre-exposed flies, but it did not mediate hyperactivity. As none of the Trp residues of the triad are included in this construct, this result raises further difficulties with the RPM as mediated by the triad. GFP is capable of absorbing blue photons and may trigger an electron transfer [49] so it could be that a GFP-mediated transfer to the CRY-C-terminus required by the RPM is mimicking the wild-type CRY response to EMF, albeit somewhat weakly. Such a model would require the GFP-CRY-CT peptide to have a FAD binding pocket, which is unlikely. Alternatively if there is no electron transfer between GFP and CT, then perhaps the CRY-CT is actually the effector for EMFs and represents the domain capable of transmitting the magnetic information by interactions with downstream molecules not yet identified. This would require another light-sensing molecule because the isolated CRY-CT would not have this ability. Such a model would have the CRY-CT mediating the period shortening EMF phenotype via this unknown light-sensor and disrupting interactions with downstream clock molecules, TIM, JETLAG and RAMSHACKLE [40], [41]. The N-terminal could mediate hyperactivity, perhaps via dCRY's known role in mediating light-dependent neuronal firing [50]. However, even though we have demonstrated that a mutation of one of the Tryptophans forming the Trp-triad is not sufficient to abolish the response, we cannot rule out that the Trp-triad is not required for the RPM without simultaneously mutating all three Trp residues.

Finally, of the two hCRYs, both of which have conserved N-terminals but diverged C-terminals compared to dCRY, expression of hCRY2 exhibited the EMF-induced hyperactivity even though neither hCRY responded to LL by increasing period. This result suggests that the C-terminal of hCRYs cannot mediate the downstream events required for period lengthening, which requires interactions with CRYs known Drosophila clock partners. However, the hyperactivity phenotype generated by hCRY2 must require a different downstream pathway that requires the more conserved N-terminal sequences. At the primary sequence level, hCRY2 is only marginally more similar to dCRY than hCRY1 (40.4% v 39.4%) in the N-terminal 500 residues, but whether this translates to more similarity in functional features of protein structure to dCRY is not known [42]. Given hCRY2's responsiveness to an EMF in flies, we subsequently examined whether a circadian assay in mouse SCN slices mediated by the endogenous type 2 mCRY1 and mCRY2 could also respond to EMFs. We were unable to demonstrate any significant effects using a number of different field intensities, in both the presence and absence of suitable illumination for CRY photoactivation. These results suggest that mCRY1 and mCRY2 are not photosensitive during the period that they are active as repressors, at least in the context of SCN neurons. There is some debate concerning the photosensitivity of vertebrate CRYs, which can show photoreduction *in vitro* [16]. Indeed, as mentioned earlier, hCRY2 shows a photosensitivity in both the conditioning [25] and our hyperactivity assay (but not in our period-lengthening LL assay), so within a *Drosophila* cellular environment, mammalian CRYs can retain light responses. Within the SCN environment, however, the endogenous mammalian CRYs show no evidence for direct sensitivity to light or EMF. As light information from the retina is transmitted to the SCN by the retinohypothalamic tract [51], perhaps the use of mouse retina, in which CRYs are also expressed at high levels may provide a more appropriate cellular milieu in which to study putative mammalian CRY-mediated responses to EMF.

In conclusion, our results have revealed that under stringently controlled conditions, circadian locomotor behavior can be used to detect two robust CRY-dependent responses to very low frequency EMFs in *Drosophila*. Our results cast further doubt on the RPM for mediating CRY EMF responses in its conventional form via the Trp triad, yet our results with 500 nm resonate with the antagonistic hypothesis, providing further support for the RPM. New putative radical partners have recently been hypothesised such as ascorbic acid [24], so while the RPM retains its validity, it is not yet clear what is the identity of all the essential players. Our future work will aim to identify the neurons and the associated molecular mechanisms that are responsible for these intriguing EMF-mediated phenotypes.

## Methods

### *Drosophila* strains

Flies were raised at 25°C on standard yeast-maize medium under a light-dark (LD 12∶12) cycle. All strains, mutants, GAL4 and UAS transgenes were backcrossed into a *w^1118^* background for 5–7 generations. *UASmychCRY1/2* and *UAScryW342F* were obtained from Steven Reppert (UMass). *timGAL4*, *UAScry24b* [11], *UASHAcry* and *UAScryΔ14.6* have been described elsewhere [26]. *UAS-GFP-C-terminal-CRY (UASGFPcryCT)* flies were crossed into a *cry^02^* background, using standard balancing techniques.

#### *UASGFPcryCT* cloning

This chimeric *cry* construct contains the C-terminal CRY residues 491–542 fused downstream of the *GFP* gene with an N-terminus tagged with Strep(II). This was generated by amplifying the GFP sequences using a forward primer *(primer-Af)* containing a start codon and the Strep(II) tag and a reverse primer possessing the relevant GFP sequence plus an additional stretch of bases complementary to the *cry* C-terminal sequence. A second amplification used a forward primer encoding a tract of complementary GFP nucleotides and the start of the *cry*- C-terminus with the reverse primer *(primer-Br)* completing the *cry* sequences plus stop codons to terminate translation. The products of the two amplifications were added together after gel-extraction with *primer-Af* and *primer-Br* to generate the chimeric construct. This was sequenced to check for errors before being inserted into pUAST and outsourced for injection (BestGene, CA, USA).

#### Behavioral analyses

Circadian locomotor activity was recorded with *Drosophila* Trikinetics Monitors (Waltham, MA) and analysed using spectral analysis and autocorrelograms [52]. To test the effects of EMF on the free-running circadian period of locomotor activity, we used a modified version of the Schuderer apparatus [31], which consists of two independent double-wrapped coils [53] placed inside two μ-metal boxes within a commercial incubator. The shielded, four quadratic Helmholtz coil systems produce a homogenous, linearly polarized *B* field (static or oscillating) with perpendicular orientation to the horizontal plane of the Trikinetics monitors (Figure S6, or the Petri dishes carrying the SCN slices, Figure S4A). Each coil is formed with a pair of wires with the current passing in the same direction through both wires for EMF exposure but in opposite directions to provide a sham exposure condition. A PC randomly selects which of the two chambers receives either the EMF or the sham exposure so the operator is blind to which is the experimental chamber. For the fly experiments we initially chose a 300 µT EMF, the intensity at which the maximal responses had been previously observed [21], oscillating at 3 Hz and in constant blue light (LL) at an intensity of 0.25 µWcm^−2^ (LED wavelength 450 nm, 40 nm broad range, RS Component). This LL intensity was operationally selected because 60% of flies remained rhythmic under these conditions so any putative effects of EMF on rhythmicity could be observed in both directions (Figure S7A). In addition, the free-running period of the rhythmic flies in dim blue light was 27.5±0.6 h compared to 24.1±0.4 h in DD (p<0.01, Figure S7B). For the 500 nm experiment the same light intensity was used.

One to three day old flies were first entrained at 25°C in the apparatus under a LD12∶12 cycle for three days using white light, before being pre-exposed to continuous dim blue light for seven days, followed by exposure to an EMF or sham for a further eight days under the same blue lighting conditions. Experiments were performed using a static field 3 Hz, 50 Hz each at 300 µT, and also at 90 µT and 1 mT at 3 Hz. Under the RPM, the effect of a superimposed EMF should not be different for static or extremely low frequency fields at the same field intensity, since the oscillations of the field are longer by several orders of magnitude than the radicals' lifetime, which is in the order of microseconds [1]. We observed that under 0.25 µWcm^−2^ a 50 Hz oscillating field exposure led to a rate of arrhythmicity in the flies well above 50% and so we reduced the blue light intensity to 0.09 µWcm^−2^. The 50 Hz EMF interfered with the circuit for the LEDs causing them to flicker and thereby raising their effective intensity. A radiometer (ILT1400 Lot Oriel) was not able to detect any flickering under static or 3 Hz EMF.

The period was determined during the pre-exposure and during the EMF or sham exposure. Statistical analyses were performed on flies that were rhythmic throughout the experiment, however for some experiments, especially when only a few flies were rhythmic both before and after the exposure, all flies that were rhythmic either before or after the exposure were included in the analysis. General activity levels were calculated for every 30 min bin regardless of period, but only rhythmic flies were included.

### dCRY antibody and Western blots

A dCRY anti-serum was generated in guinea-pig against the N-terminal 188 residues of *Drosophila* CRY fused to GST. In three diagnostic CRY tests, western blots of fly heads revealed that the reagent detected a high level of endogenous CRY from wild-type flies maintained in darkness, which was dramatically reduced in the *cry^b^* nearly-null mutant [10], in the *cry^02^* mutant (Figure 7A) as well as in wild-type flies maintained in both under normal laboratory lighting and in constant dim blue light (Fig. 7 sham condition, [11]). For the EMF or sham blots, flies were harvested after 5 days under constant dim blue light and constant darkness (DD) controls were generated by using flies in vials wrapped in aluminium foil and placed inside the same boxes so exposed to the same EMF or sham conditions. A pool of 100 heads, collected at ZT14, was homogenized in 1.5 volume of extraction buffer (20 mM Hepes, pH 7.5, 100 mM KCl, 2.5 mM EDTA, pH 8, 5% glycerol, 0.5% Triton X-100, 1 mM DTT, complete protease inhibitors tablets from Roche). After quantification *via* Bradford (Sigma) assay, proteins were loaded on a 10% SDS-page and transferred to Nitrocellulose Membrane (GE HealthCare). The following primary antisera were used: mouse Guinea Pig anti-CRY (1∶1,000) and mouse anti-HSP70 (Sigma, 1∶50,000). Secondary horseradish peroxidase–conjugated antisera were goat anti-guinea pig (ABCam Ltd, 1∶10,000) and goat anti-mouse (Sigma, 1∶6,000). Signals were obtained by chemiluminescence (ECL, GE HealthCare) and quantified with GelAnalyser 2010 (GelAnalyser.com, Dr Istvan Lazar). Three biological replicates with three technical replicates (ca 30 heads each) were performed.

Western blots on the *UAS-GFP-C-terminal-CRY*, *UAScryW342F and UASmychCRY1* crossed to *timGAL4* were performed as followed: Ten to fifteen flies were kept in DD for 3 days and during the fourth subjective night (ZT 20–22) were collected. Proteins were extracted as described above. The following primary antisera were used: mouse Guinea Pig anti-CRY (1∶1,000, used for *UAS-GFP-C-terminal-CRY*, *UAScryW342F*), mouse anti-MYC (Invitrogen, 1∶3000, used for *UASmychCRY1*), mouse anti-HSP70 (Sigma, 1∶50,000) and mouse anti-TUBα (Sigma, 1∶10000, used for *UASmychCRY1*). Secondary horseradish peroxidase–conjugated antisera were goat anti-guinea pig (ABCam Ltd, 1∶10,000) and goat anti-mouse (Sigma, 1∶6,000). Signals were obtained by chemiluminescence (ECL, GE HealthCare) and quantified with GelAnalyser 2010 (GelAnalyser.com, Dr Istvan Lazar). Three biological replicates with three technical replicates (ca 30 heads each) were performed.

### Mouse SCN slices

All animal work carried out in these studies was licensed under the UK Animals (Scientific Procedures) Act 1986, with Local Ethical Review by the MRC. Sacrifice was by cervical dislocation. Wild type (WT) *Per2:Luc* mice, generated by J. Takahashi (University of Texas Southwestern Medical Center, Dallas), were housed under a 12 h light∶12 h dark cycle. Brains were removed from pups (P7–P10) and SCN organotypic slices were prepared as previously described [54]. After at least 7 days, SCN slices were transferred to a photon multiplier tube assembly (PMT) for bioluminescence recordings.

### EMF exposure for SCN slices

SCN slices were incubated in a Schuderer apparatus-based system, within a light-tight incubator at 37°C, with fibre-optic transmission of bioluminescence signals to a PMT assembly housed outside the incubator to avoid interference with the EMF (Figure S4A). For light exposure, SCN slices were exposed to either 405 nm (blue) or 625 nm (red) light from high-power LEDs (Thorlabs, UK) at 1 µW/cm^2^ coupled to the fibre-optics used for bioluminescence transmission. Automated control of LEDs and PMT allowed a cycle of intermittent light and bioluminescent recordings consisting of 23 min light exposure, 30 s delay, 6 min PMT capture, 30 s delay, providing bioluminescence data acquisition every 30 min.

### Statistical analyses

Statistical analyses of *Drosophila* locomotor rhythms were performed using spectral analysis implemented in the custom-written BeFly! package [52], [55]. Further analyses were carried out using GraphPad Prism version 6.00 for Windows, (GraphPad *Software*, La Jolla California USA, www.graphpad.com) and STATISTICA (data analysis software system, version 8.0 StatSoft, Inc. 2008, www.statsoft.com). Rhythmic bioluminescence was analysed in BioDare software (A. Millar, University of Edinburgh, UK). A repeated-measure two-way ANOVA was used to test for significant influences of magnetic field exposure and order of field application on circadian period. Period error (a measure of cycle to cycle variability) and relative amplitude error (RAE, an index of the rhythmic coherence of the slice) of SCN bioluminescence was also analysed.

## Supporting Information

Figure S1 Period changes are not caused by mechanical vibration. A. When one of the two fans was unplugged from the mains to reduce vibration in one chamber, there were no differences observed in period under dim blue light between wild-type flies in the two chambers (F_(1,31)_ = 0.17, p = 0.68, N = 16 for both conditions) B. When both fans were plugged in for a sham exposure condition, there were no differences observed in period under dim blue light (F_(1, 36)_ = 1.7, p = 0.27, N 18 and 19). Mean ± sem.
(TIFF)

Figure S2 Representation of CRY variants used. Bold residues symbolise the position of the mutation: the red-circled “W” indicates that the Trp342 has been substituted with Phe. Red plus green residues indicate the residues used for making the *GFPcryCT* construct whereas green shows the residues deleted in CRYΔ. red zig-zag represents H-alpha and other helices, green arrows are E-beta strand or bridge and blue bars show C-coil.
(TIF)

Figure S3 Light responsiveness of CRY variants. Mean ± sem and Table S1 shows the periods and Ns. A *tim>cryW342F; cry^02^* flies still show a light responsiveness (F_(1,35)_ = 3.30, p<0.05) B *tim>cryΔ;cry^02^* overexpressing *cryΔ* leads to a period-lengthening in dim blue LL compared to DD. C *tim>cryCT; cry^02^* flies show light responsiveness (F_(2,74)_ = 32.29, p<0.001) (*post hoc* *p<0.5, **p<0.01, ***p<0.001). D Western blots of *tim>cryW342F; cry^02^*, *tim>cryCT; cry^02^*and *tim>hCRY1;cry^02^* fly heads using anti-dCRY and anti-MYC (for *hCRY1* only) showing that the constructs are expressed and detectable.
(TIFF)

Figure S4 SCN exposure to EMF. (A) Schematic representation of exposure system. Within the incubator are two μ-metal shield boxes that hold up to four SCN each. EMF is generated within the μ-metal shield chambers and SCN bioluminescence is transmitted to a PMT assembly house outside the incubator. Arrows indicate air flow. There are 2 chambers within the incubator holding 4 samples each. (B) Representative recording of Per2::Luc bioluminescence from a WT SCN explant. Shading indicates exposure to an oscillating 50 Hz 300 µT field. (C–E) Paired circadian periods of slices in sham and exposure conditions (n = 10 for each exposure strength). (F–H) Grouped data of period (F), period error (G) and relative amplitude error (H) of SCN explants under exposure to different strength, oscillating 50 Hz fields. Error bars = +SEM, n = 10 for each field strength, except n = 5 for 150 µT. There are no significant differences between groups.
(TIFF)

Figure S5 No EMF-induced effects by blue or red light on SCN. (A) Representative recording of Per2::Luc bioluminescence from SCN explants under intermittent blue light. Shading indicates duration of field and light exposure. (B) Intermittent blue light exposure alone does not have any effect on the period of SCN slices. (C, D) Paired circadian periods of slices in sham and exposure conditions under blue or red intermittent light. (E-F) Period error and (G-H) relative amplitude error of SCN explants under exposure to different strength oscillating 50 Hz fields. Hatched bars = field exposure, clear bars = sham exposure, +SEM. There are no significant differences between groups, n = 12 for each condition in C-H.
(TIF)

Figure S6 Schematic representation of the Schuderer Apparatus for flies [31]. The blue arrows represent the air flow through the chambers.
(TIFF)

Figure S7 Rhythmicity of wild-type under different intensities of constant blue light. (A) % of rhythmic CS under different blue light intensities. Heterogeneity χ^2^_(4)_ = 16.19, p = 0.0028. (B) Period lengthening of CS flies under different blue light intensities. F_(4,53)_ = 6.79, p<0.001. 0.16 µWcm^−2^ = 26.80±0.35, N = 14, 0.18 µWcm^−2^ = 26.97±0.44, N = 16; 0.25 µWcm^−2^ = 27.53±0.64, N = 12; 0.40 µWcm^−2^ = 29.04±1.10, N = 8; DD = 24.1±0.40, N = 8. (*post-hoc* *p<0.05, **p<0.01, ***p<0.001). Mean ± sem.
(TIFF)

Table S1 Summary of circadian behavior.
(TIFF)

Table S2 Summary of hyperactivity.
(TIFF)
