# Supplementary figures and images for: Genetic Analysis of Circadian Responses to Low Frequency Electromagnetic Fields in Drosophila melanogaster
Source: PLoS Genet. 2014 Dec 4;10(12):e1004804. doi: 10.1371/journal.pgen.1004804 (PMC4256086; doi:10.1371/journal.pgen.1004804)

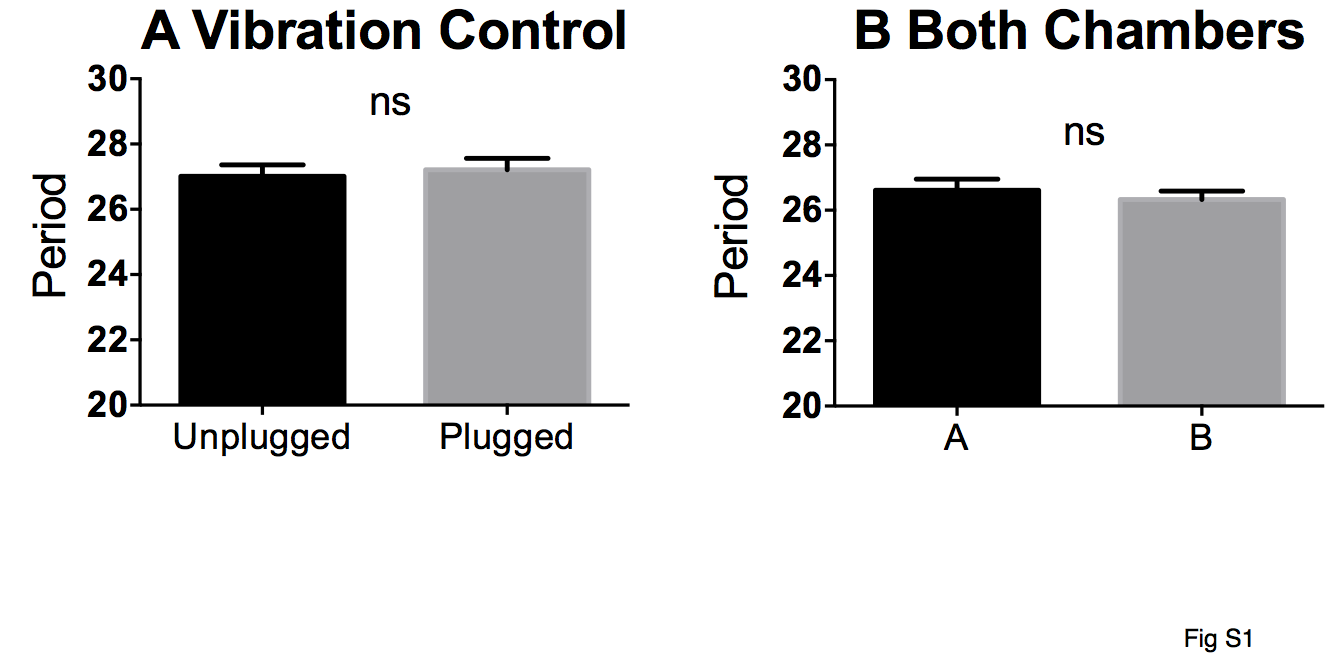

Supplement: Figure S1 — Period changes are not caused by mechanical vibration. A. When one of the two fans was unplugged from the mains to reduce vibration in one chamber, there were no differences observed in period under dim blue light between wild-type flies in the two chambers (F(1,31) = 0.17, p = 0.68, N = 16 for both conditions) B. When both fans were plugged in for a sham exposure condition, there were no differences observed in period under dim blue light (F(1, 36) = 1.7, p = 0.27, N 18 and 19). Mean ± sem. (TIFF) [file pgen.1004804.s001.tiff]

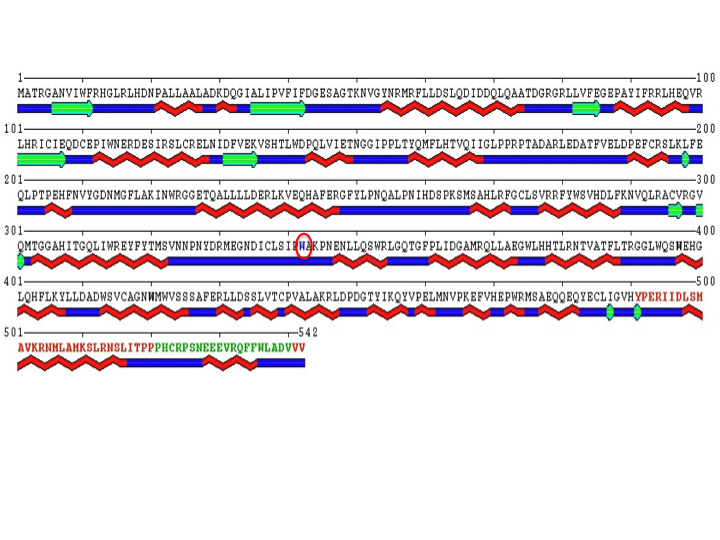

Supplement: Figure S2 — Representation of CRY variants used. Bold residues symbolise the position of the mutation: the red-circled “W” indicates that the Trp342 has been substituted with Phe. Red plus green residues indicate the residues used for making the GFPcryCT construct whereas green shows the residues deleted in CRYΔ. red zig-zag represents H-alpha and other helices, green arrows are E-beta strand or bridge and blue bars show C-coil. (TIF) [file pgen.1004804.s002.tif]

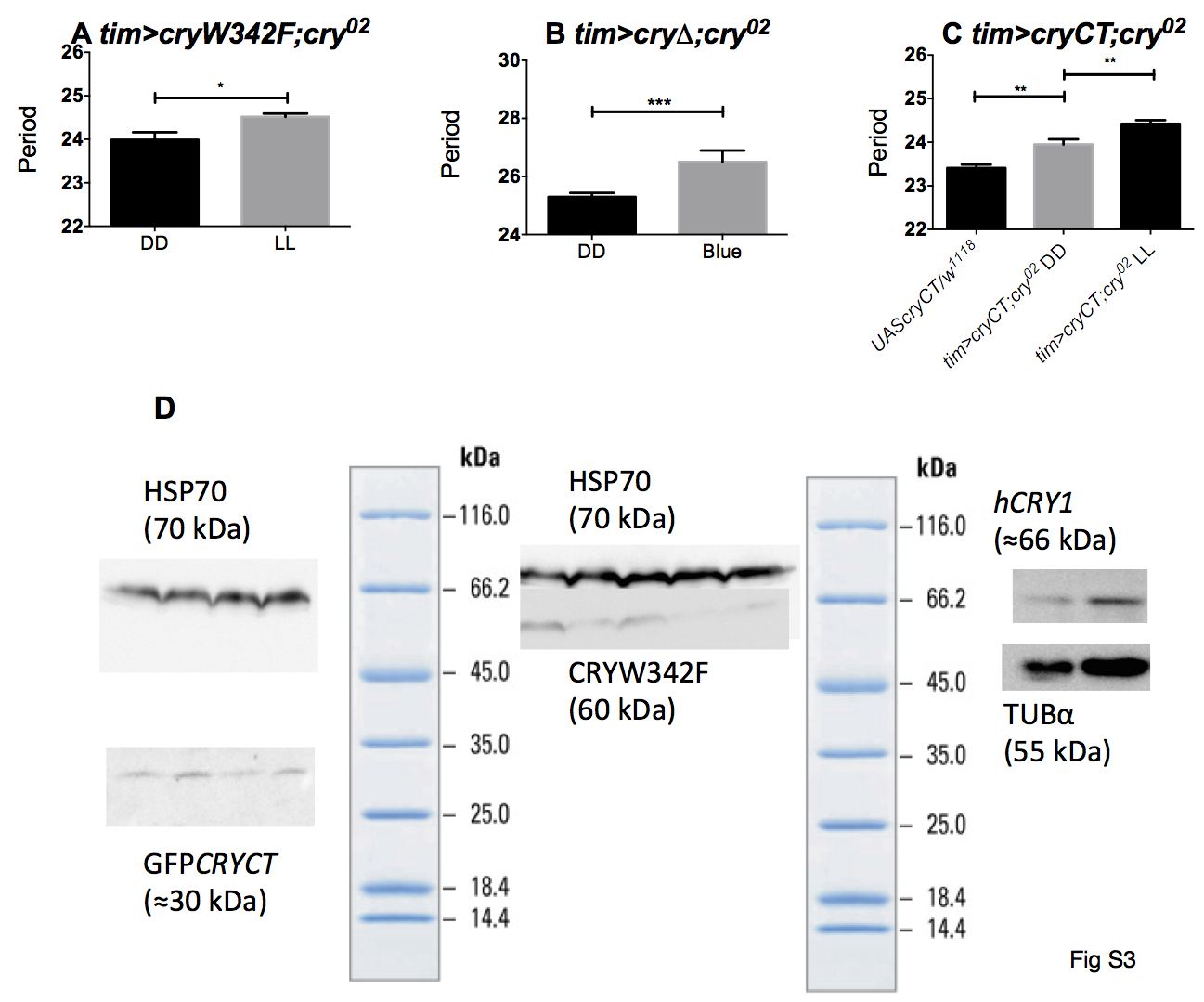

Supplement: Figure S3 — Light responsiveness of CRY variants. Mean ± sem and Table S1 shows the periods and Ns. A tim>cryW342F; cry02 flies still show a light responsiveness (F(1,35) = 3.30, p<0.05) B tim>cryΔ;cry02 overexpressing cryΔ leads to a period-lengthening in dim blue LL compared to DD. C tim>cryCT; cry02 flies show light responsiveness (F(2,74) = 32.29, p<0.001) (post hoc *p<0.5, **p<0.01, ***p<0.001). D Western blots of tim>cryW342F; cry02, tim>cryCT; cry02and tim>hCRY1;cry02 fly heads using anti-dCRY and anti-MYC (for hCRY1 only) showing that the constructs are expressed and detectable. (TIFF) [file pgen.1004804.s003.tiff]

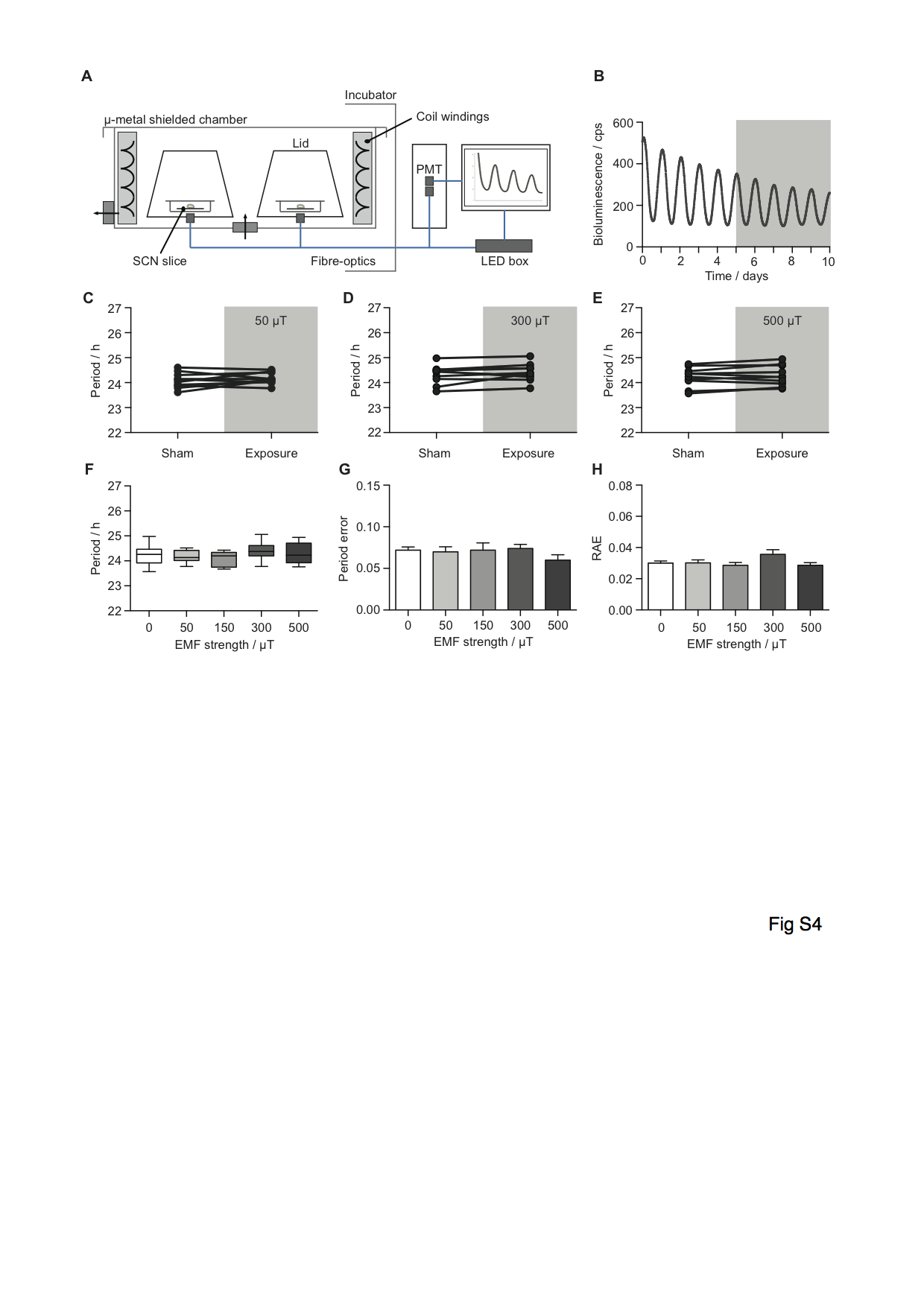

Supplement: Figure S4 — SCN exposure to EMF. (A) Schematic representation of exposure system. Within the incubator are two μ-metal shield boxes that hold up to four SCN each. EMF is generated within the μ-metal shield chambers and SCN bioluminescence is transmitted to a PMT assembly house outside the incubator. Arrows indicate air flow. There are 2 chambers within the incubator holding 4 samples each. (B) Representative recording of Per2::Luc bioluminescence from a WT SCN explant. Shading indicates exposure to an oscillating 50 Hz 300 µT field. (C–E) Paired circadian periods of slices in sham and exposure conditions (n = 10 for each exposure strength). (F–H) Grouped data of period (F), period error (G) and relative amplitude error (H) of SCN explants under exposure to different strength, oscillating 50 Hz fields. Error bars = +SEM, n = 10 for each field strength, except n = 5 for 150 µT. There are no significant differences between groups. (TIFF) [file pgen.1004804.s004.tiff]

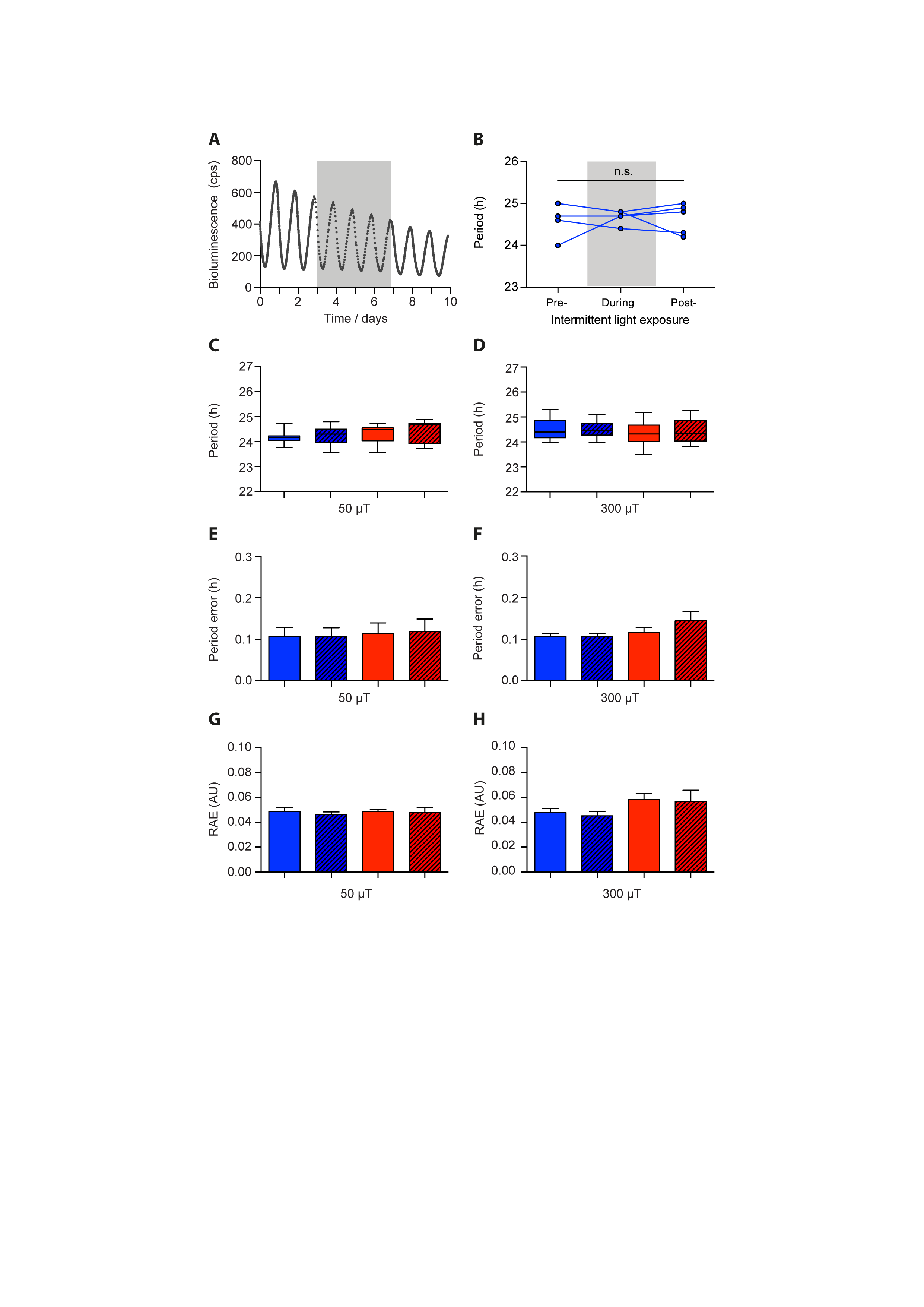

Supplement: Figure S5 — No EMF-induced effects by blue or red light on SCN. (A) Representative recording of Per2::Luc bioluminescence from SCN explants under intermittent blue light. Shading indicates duration of field and light exposure. (B) Intermittent blue light exposure alone does not have any effect on the period of SCN slices. (C, D) Paired circadian periods of slices in sham and exposure conditions under blue or red intermittent light. (E-F) Period error and (G-H) relative amplitude error of SCN explants under exposure to different strength oscillating 50 Hz fields. Hatched bars = field exposure, clear bars = sham exposure, +SEM. There are no significant differences between groups, n = 12 for each condition in C-H. (TIF) [file pgen.1004804.s005.tif]

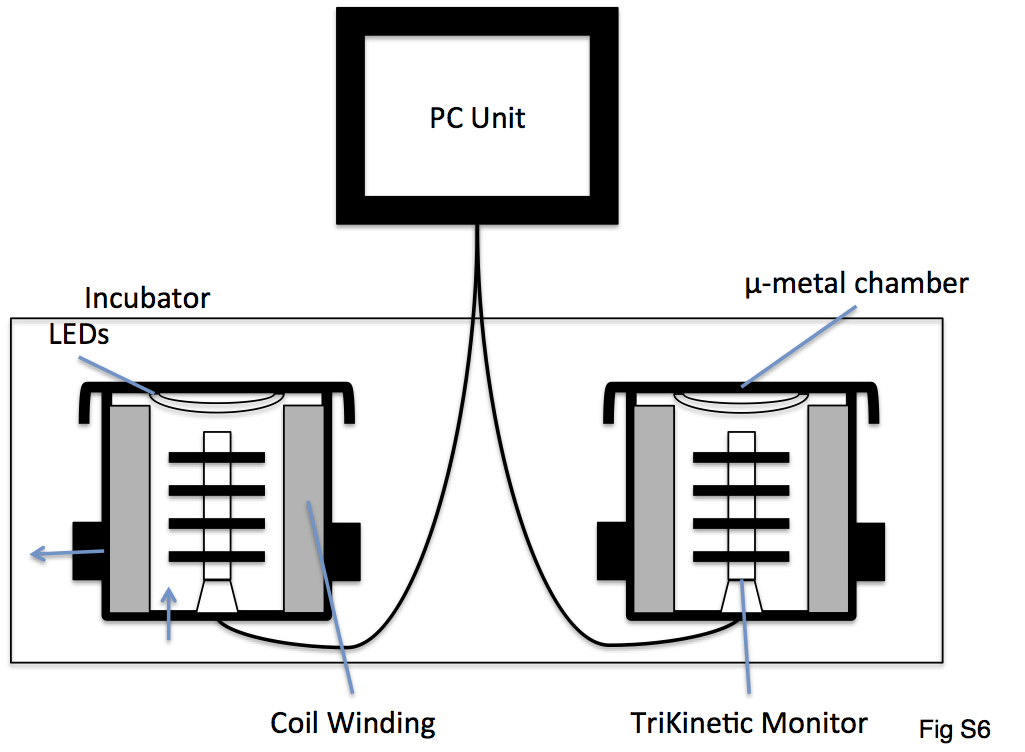

Supplement: Figure S6 — Schematic representation of the Schuderer Apparatus for flies [31]. The blue arrows represent the air flow through the chambers. (TIFF) [file pgen.1004804.s006.tiff]

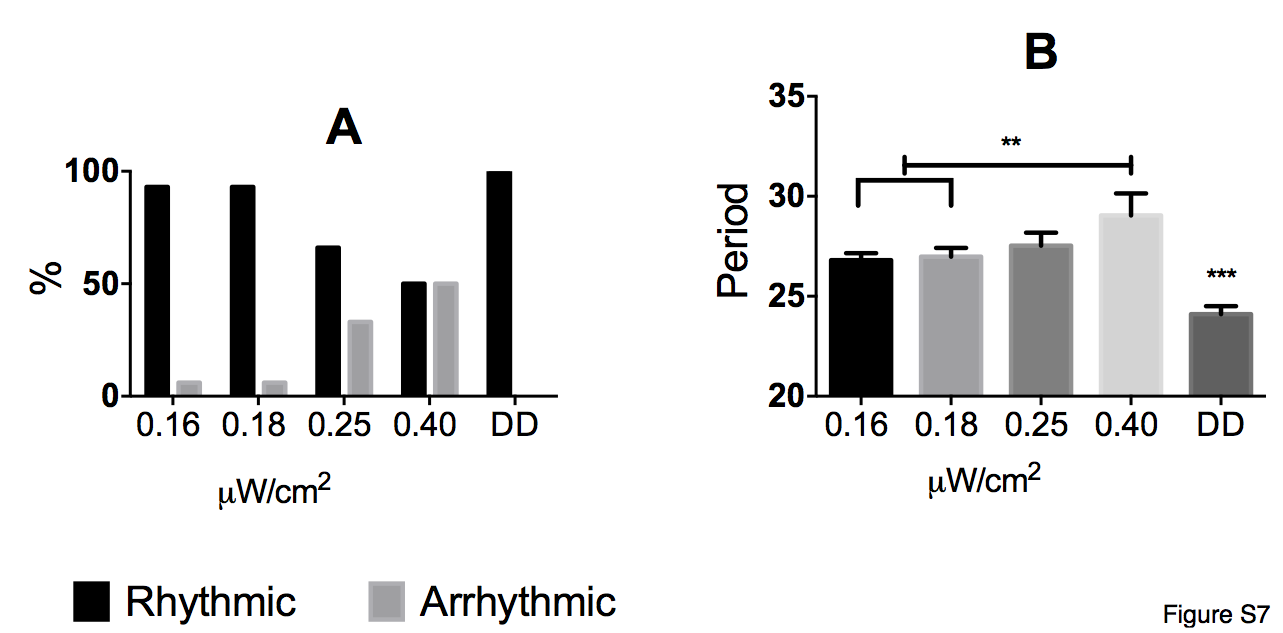

Supplement: Figure S7 — Rhythmicity of wild-type under different intensities of constant blue light. (A) % of rhythmic CS under different blue light intensities. Heterogeneity χ2 (4) = 16.19, p = 0.0028. (B) Period lengthening of CS flies under different blue light intensities. F(4,53) = 6.79, p<0.001. 0.16 µWcm−2 = 26.80±0.35, N = 14, 0.18 µWcm−2 = 26.97±0.44, N = 16; 0.25 µWcm−2 = 27.53±0.64, N = 12; 0.40 µWcm−2 = 29.04±1.10, N = 8; DD = 24.1±0.40, N = 8. (post-hoc *p<0.05, **p<0.01, ***p<0.001). Mean ± sem. (TIFF) [file pgen.1004804.s007.tiff]

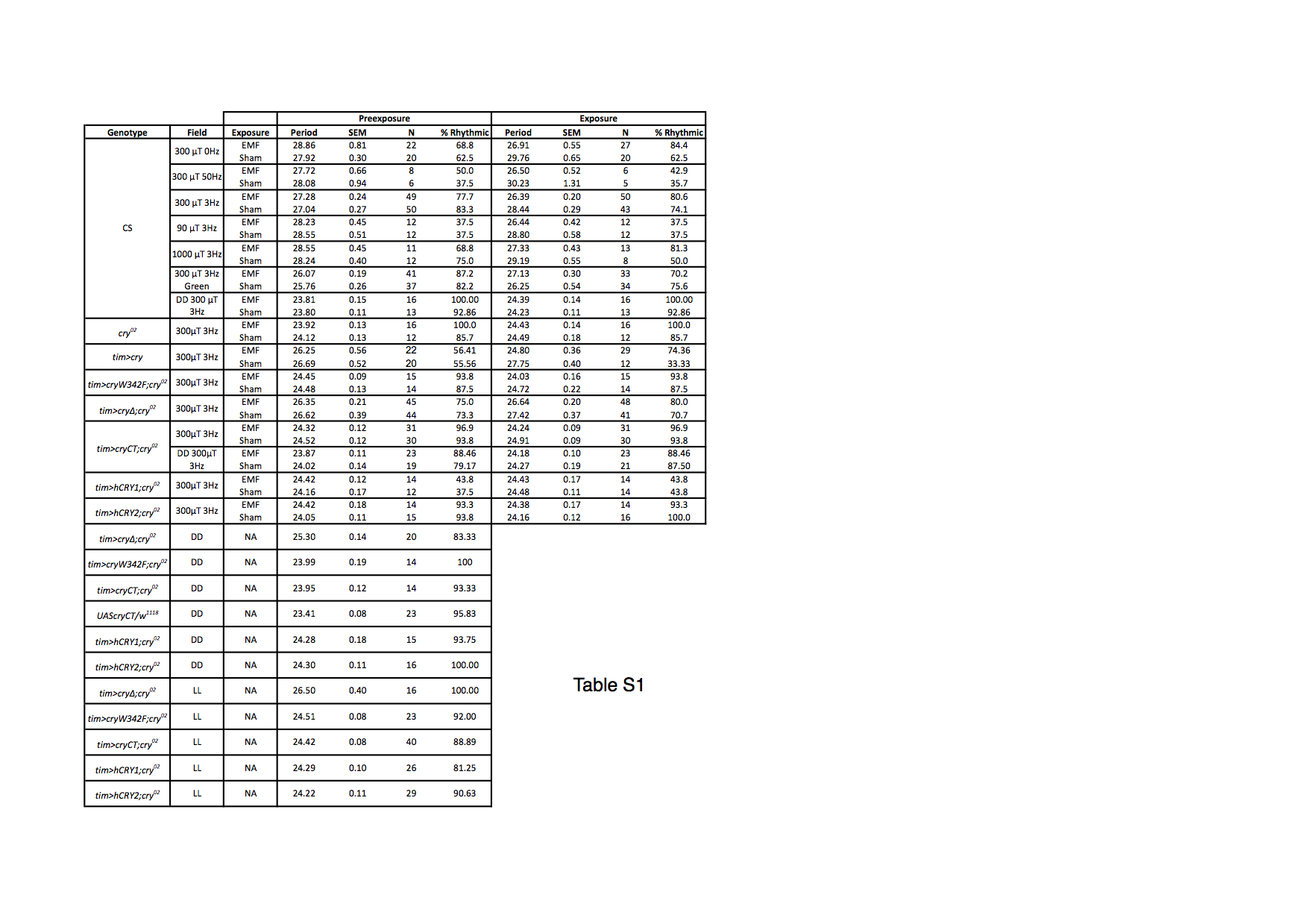

Supplement: Table S1 — Summary of circadian behavior. (TIFF) [file pgen.1004804.s008.tiff]

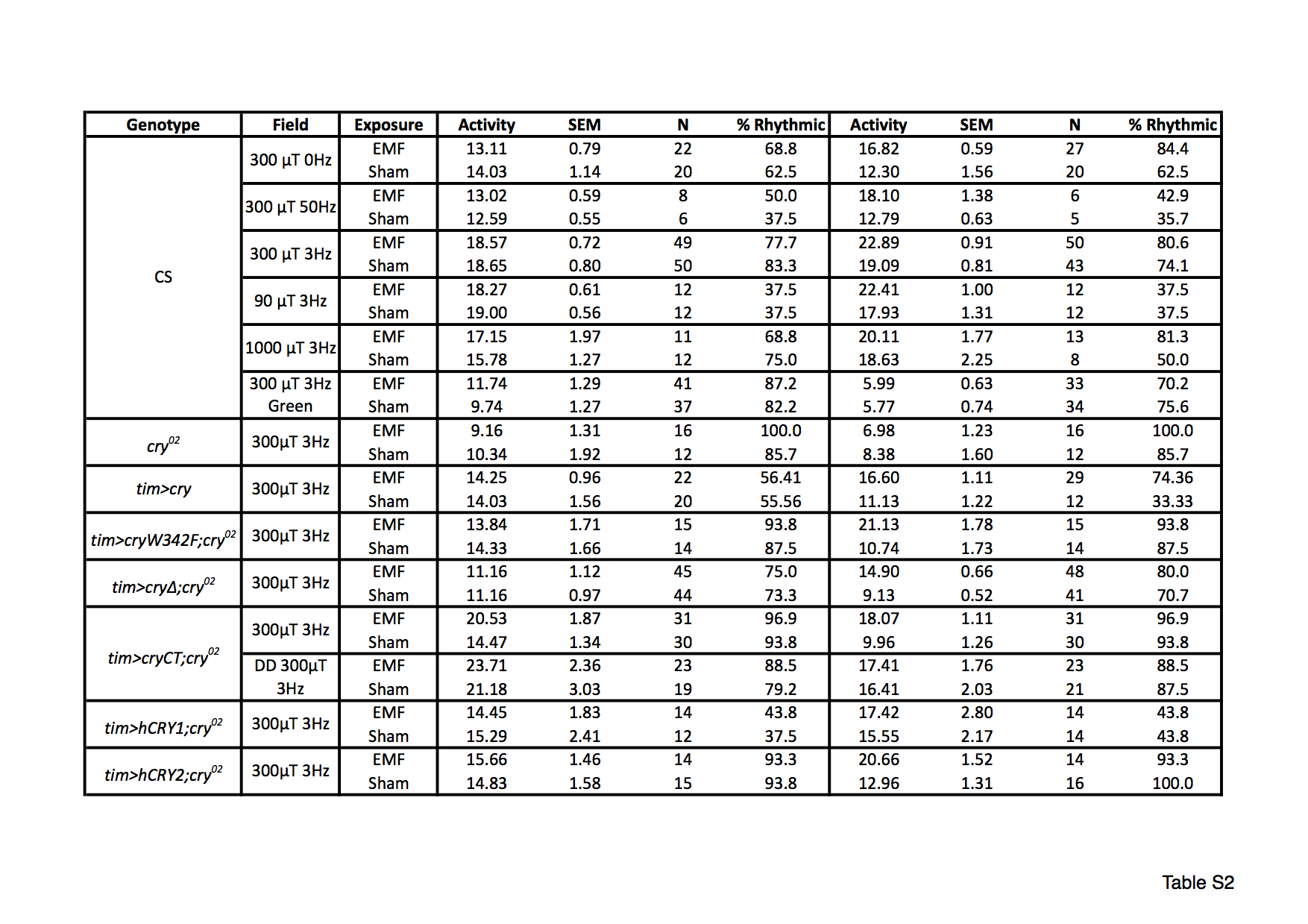

Supplement: Table S2 — Summary of hyperactivity. (TIFF) [file pgen.1004804.s009.tiff]
